# Impact of cellular folate status and epidermal growth factor receptor expression on BCRP/ABCG2-mediated resistance to gefitinib and erlotinib

**DOI:** 10.1038/sj.bjc.6604980

**Published:** 2009-03-10

**Authors:** C Lemos, I Kathmann, E Giovannetti, C Calhau, G Jansen, G J Peters

**Affiliations:** 1 Department of Biochemistry (U38-FCT), Faculty of Medicine, University of Porto, Porto 4200-319, Portugal; 2 Department of Medical Oncology, VU University Medical Center, Amsterdam 1081 HV, The Netherlands; 3 Department of Rheumatology, VU University Medical Center, Amsterdam 1081 HV, The Netherlands

**Keywords:** BCRP, erlotinib, folate, gefitinib, drug resistance

## Abstract

The effect of folate status on breast cancer resistance protein (BCRP)-mediated drug resistance to epidermal growth factor receptor (EGFR)-targeted drugs, such as gefitinib and erlotinib, was investigated in two human colon cancer cell lines, WiDr and Caco-2, of which the latter displayed greater sensitivity to these drugs due to high EGFR expression. Caco-2 LF/LV cells, growing under low-folate (LF) conditions, showed increased BCRP protein expression compared with the high-folate (HF) counterpart, which was associated with 1.8-fold resistance to gefitinib. Of note, the BCRP-specific inhibitor Ko143 completely reverted this phenotype. WiDr LF cells also showed slightly increased BCRP expression compared with the HF cells, but no differences in gefitinib sensitivity were observed. Both Caco-2 LF/LV and WiDr LF cells showed 2.4- and 2.3-fold resistance to erlotinib, respectively, compared with their HF counterparts, which mechanistically seemed BCRP unrelated, as Ko143 had no effect on erlotinib activity. In conclusion, our data suggest that in EGFR-expressing Caco-2 cells, BCRP is one of the determinants of gefitinib resistance but not of erlotinib resistance. Beyond this, folate depletion can provoke an additional decrease in gefitinib and erlotinib activity by mechanisms dependent or independent of BCRP modulation.

The epidermal growth factor receptor (EGFR) signalling pathway has an important function in tumour development and progression. In human solid tumours, receptor overactivation and/or dysregulation, and concomitant activation of downstream routes, promotes cell proliferation and survival, angiogenesis, metastatic spread and inhibition of apoptosis. Thus, targeting the EGFR has become an important approach in cancer therapy (Ciardiello and Tortora, 2001; Mendelsohn and Baselga, 2003). Among the several potential strategies for targeting this receptor, the development of low-molecular-weight EGFR tyrosine kinase inhibitors (TKIs), such as gefitinib (Iressa) and erlotinib (Tarceva), has revealed promising results (Ciardiello and Tortora, 2001; Janmaat and Giaccone, 2003; Mendelsohn and Baselga, 2003). These orally active compounds are reversible competitors with ATP for binding to the intracellular protein tyrosine kinase domain of the receptor, thereby blocking receptor activation and downstream signalling (Ciardiello and Tortora, 2001; Janmaat and Giaccone, 2003; Mendelsohn and Baselga, 2003; Steeghs *et al*, 2007).

The breast cancer resistance protein (BCRP/ABCG2) is a member of the major family of ATP-binding cassette (ABC) transporters, which also includes P-glycoprotein (ABCB1) and the multidrug resistance protein (ABCC) family (Borst and Elferink, 2002). Similar to other members of the family, BCRP overexpression is commonly associated with multidrug resistance in cancer cells, as it has the ability to extrude a variety of anticancer drugs that appear to be structurally and mechanistically unrelated. The spectrum of anticancer drugs effluxed by BCRP includes mitoxantrone, camptothecins, anthracyclines, flavopiridol and antifolates (Assaraf, 2006; Robey *et al*, 2007).

Some recent studies have shown that gefitinib is an inhibitor and a substrate for BCRP (Elkind *et al*, 2005; Nakamura *et al*, 2005). Nonetheless, contradictory results have been reported. Nakamura *et al* (2005) reported that gefitinib was able to reverse drug resistance through inhibition of drug efflux in three multidrug-resistant cancer cell lines overexpressing BCRP. However, the same authors demonstrated that gefitinib was not a substrate for BCRP. In contrast, Elkind *et al* (2005) showed that BCRP can actively pump gefitinib out of A431 cells expressing wild-type BCRP. The apparent discrepancy between these studies is, most likely, due to the selected concentrations of gefitinib used. As it was recently shown by Li *et al* (2007), gefitinib is transported by BCRP at low concentrations (eg, 0.1 and 1 *μ*M); however, at higher drug concentration (eg, 10 *μ*M) gefitinib is no longer transported by BCRP and it exhibits an inhibitory effect on the transporter (Li *et al*, 2007). Several other TKIs were shown to interact with BCRP, such as canertinib (Erlichman *et al*, 2001), imatinib (Burger *et al*, 2004; Houghton *et al*, 2004; Jordanides *et al*, 2006; Brendel *et al*, 2007) and nilotinib (Brendel *et al*, 2007). Also for erlotinib some interactions with BCRP have been described. A preliminary study suggested that erlotinib is also a substrate of BCRP (Marchetti *et al*, 2008). In fact, it has been shown that BCRP is able to transport erlotinib at low concentrations (0.1–1 *μ*M) (Li *et al*, 2007). In addition, it was shown that erlotinib at higher drug concentrations is a BCRP inhibitor (Shi *et al*, 2007). It has been hypothesised that the phosphorylation of EGFR may activate Akt phosphorylation, which may subsequently affect the function and localisation of BCRP (Lemos *et al*, 2008a). Also single nucleotide polymorphisms (SNPs) in the *ABCG2* gene might affect the protein expression and function of the transporter (Yanase *et al*, 2006). In particular, the BCRP C421A SNP has been associated with decreased levels of protein expression and drug resistance (Imai *et al*, 2002), which might have important impact in the efficacy/toxicity of some BCRP drug substrates such as gefitinib (Cusatis *et al*, 2006; Li *et al*, 2007).

Apart from anticancer drugs, BCRP has also the ability to transport natural compounds such as folates (Chen *et al*, 2003). In this context, recent studies have shown that folate status might have significant implications in functional activity of BCRP (Ifergan *et al*, 2004; Lemos *et al*, 2008b). From this perspective, the aim of this study was to investigate the effect of cellular folate status on BCRP-mediated resistance to the EGFR TKIs gefitinib and erlotinib.

## Materials and methods

### Chemicals

Folic acid (FA), leucovorin (LV) and sulphorhodamine B (SRB) were obtained from Sigma (St Louis, MO, USA). Protease inhibitor cocktail was obtained from Roche Diagnostics (Mannheim, Germany). Ko143 was generously provided by Professor GJ Koomen, University of Amsterdam, Amsterdam, the Netherlands. Erlotinib was a gift from Roche Pharmaceuticals (Mannheim, Germany) and gefitinib from AstraZeneca (Macclesfield, UK).

### Cell culture

The human colon adenocarcinoma cell lines Caco-2 and WiDr, with constitutive BCRP expression, were cultured in RPMI 1640 medium (Cambrex Bioscience, Verviers, Belgium) containing 2.3 *μ*M FA (HF, high folate), supplemented with 10% fetal calf serum (FCS; Greiner Bio-One, Frickenhausen, Germany) and 20 mM of HEPES (Cambrex Bioscience). Caco-2 LF/LV, Caco-2 LF/FA and WiDr LF, originally isolated by gradual deprivation of folates from the growth medium (van der Wilt *et al*, 1995; Lemos *et al*, 2008b), were cultured in FA-free RPMI 1640 medium (Invitrogen, Grand Island, NY, USA) supplemented with 10% dialysed FCS (Invitrogen), 20 mM of HEPES, and 1 nM LV (Caco-2 LF/LV), 1 nM FA (Caco-2 LF/FA) or 2.5 nM LV (WiDr LF).

The human breast cancer cell line with acquired resistance to mitoxantrone (MR), MCF-7/MR (Taylor *et al*, 1991), was cultured in RPMI 1640 medium containing 2.3 *μ*M FA, supplemented with 5% FCS, 2 mM L-glutamine, penicillin, streptomycin and was bimonthly pulsed with 100 nM of MR.

### Growth inhibition studies

Growth inhibition by gefitinib and erlotinib was determined with the SRB assay as described previously (Keepers *et al*, 1991). Shortly, cells were seeded in 100 *μ*l of medium, in triplicate, in 96-well flat bottom plates at a density of 8000 (Caco-2) or 5000 (WiDr) cells per well. Cells were allowed to attach for 24 h at 37°C. Attached cells were then exposed to various concentrations of drug (5–50 000 nM of gefitinib or erlotinib) in 100 *μ*l of medium. The BCRP-specific inhibitor Ko143 (Allen *et al*, 2002) was added 15 min before the drugs and it was present during the next 72 h at a concentration of 200 nM. After a drug exposure time of 72 h, cells were fixed with trichloroacetic acid, stained with SRB protein dye and optical density (OD) was measured at 492 nm. Control wells, containing cells with culture medium but no drugs, were cultured for 1 day (day 0) followed by an additional 72 h and were used to determine the control cell growth at 72 h compared to the initial number of cells (day 0 value); wells with culture medium only were used as blanks. To distinguish between cell growth inhibition and cell kill, we corrected the OD after 72 h for the mean OD observed for the control wells at the day of drug addition (day 0 value). For each experiment the results were plotted in an Excel graph and the IC_50_ value was determined. The IC_50_, defined as the drug concentration that corresponds to a reduction of cellular growth by 50% when compared with values of untreated control cells, was used as a measure of resistance.

### Western blot analysis of BCRP and EGFR expression

Total lysates were prepared in buffer containing 50 mM Tris (pH 7.6), 20% (v/v) glycerol, 5 mM DTT, 0.5% (v/v) NP-40 and 4.0% (v/v) of a protease inhibitor cocktail. Lysates were sonicated three times for 5 s with 30 s interval (MSE Soniprep 150, 4°C, amplitude 6–7) and centrifuged at 13 000 r.p.m. for 10 min at 4°C. The protein-containing supernatant was collected and protein content was determined using the Bio-Rad protein assay. In each lane of a Bio-Rad mini-gel system (Bio-Rad, Hemel Hempstead, UK) 1–40 *μ*g of proteins was loaded. For detection of BCRP a monoclonal rat anti-BCRP antibody (BXP-53; 1 : 200; 1.25 *μ*g ml^−1^) (Jonker *et al*, 2002) was used (kindly provided by Dr GL Scheffer, VU University Medical Center, Amsterdam, the Netherlands). For detection of phospho-Akt (pAkt), we used a rabbit anti-pAkt (Ser 473) antibody (Cell Signaling Technology, Danvers, MA, USA; 1 : 500) and for detection of EGFR, we used a rabbit anti-EGFR antibody (Cell Signaling Technology; 1 : 1000). As secondary antibodies, horseradish peroxidase-conjugated rabbit anti-rat (DakoCytomation, Glostrup, Denmark; 1 : 2000) and donkey anti-rabbit (GE Healthcare, Chalfont St Giles, UK; 1 : 2000) were used. As a loading control, expression of *β*-actin was determined using an antibody against *β*-actin (clone C4 from Chemicon International, Temecula, CA, USA; 1 : 10 000; 0.01 *μ*g ml^−1^).

### Real-time LC-PCR

Total RNA was isolated from Caco-2 and WiDr cells using the RNeasy Plus Mini Kit (Qiagen, Hilden, Germany) according to manufacturer's instructions. RNA was reverse-transcribed to cDNA using random hexamers as previously described (van der Wilt *et al*, 2003).

Real-time PCR analysis was performed using the LightCycler (LC) instrument (Roche Diagnostics, Penzberg, Germany) and Hybridization Probes, essentially as described earlier (Sigmond *et al*, 2004). All samples were tested by the LightCycler FastStart DNA Master^PLUS^ HybProbe kit (Roche Diagnostics) according to manufacturer's recommendations. PCR reactions were performed in duplicate using 5 *μ*l of cDNA, which was added to 15 *μ*l of reaction mixture containing 4 *μ*l of LightCycler FastStart DNA Master^PLUS^ HybProbe ‘Master Mix’ (previously prepared by the addition of 10 *μ*l of ‘Enzyme’ to one vial of ‘Reaction Mix’), 4 *μ*l (BCRP) or 0.4 *μ*l (*β*-actin) of a mix containing forward and reverse primers and probe, and water (PCR grade), in a final volume of 20 *μ*l. The final concentration of each component in a 20 *μ*l reaction mixture was as follows: 50 mU *μ*l^−1^ Taq DNA polymerase, 200 *μ*M dNTPs and 1.5 mM MgCl_2_. For BCRP, forward primer, 5′-AGATGGGTTTCCAAGCGTTCAT; reverse primer, 5′-CCAGTCCCAGTACGACTGTGACA; and probe, 5′-6FAM-TGCTGGGTAATCCCCAGGCCTCTATAGC-TAMRA, were used at a final concentration of 400 and 200 nM, for primers and probe, respectively. For *β*-actin, forward primer, 5′-TCACCCACACTGTGCCCATCTACGA; reverse primer, 5′-CAGCGGAACCGCTCATTGCCAATGG; and probe, 5′-6FAM-ATGCCCTCCCCCATGCCATCCTGCGT-TAMRA, were used at a final concentration of 200 nM. The primers and probes used were designed using LightCycler probe design software (Roche Diagnostics).

The PCR program consisted of an initial denaturation step at 95°C for 10 min and 45 cycles of warming up until 95°C immediately followed by 15 s at 60°C. After the final cycle, the capillaries were cooled for 30 s at 40°C.

Fluorescence curves were analysed with the LC software (version LCS4 4.0.5.415). This software uses the second derivative maximum method to calculate the fractional cycle numbers where the fluorescence rises above background (crossing point, CP); that is, the point at which the rate of change of fluorescence is fastest.

The expression of BCRP was quantified relative to *β*-actin. For this purpose, standard calibration curves were made for both targets by amplifying 5 *μ*l of various dilutions of cDNA from a pool of three cell lines (CEM/7A, MCF-7/MR and SW1573 2R/120), as described above. For the standard curves, CPs were plotted *vs* log concentration for the standards. These standard curves were used to estimate the concentration of each sample.

### BCRP polymorphism

The rs2231142 polymorphism of ABCG2 was studied with TaqMan probes-based assays using the ABI PRISM 7500 instrument equipped with the Sequence Detection System version 2.0 software (Applied Biosystems, Foster City, CA, USA). Forward and reverse primers and probes (Applied Biosystems SNP Genotyping Assays products) were obtained from Applied Biosystems (C_15854163_70, TaqMan Drug Metabolism Genotyping Assays). The PCR reactions were performed using 20 ng of genomic DNA diluted in 11.875 *μ*l DNAse-RNAse free water, 12.5 *μ*l of TaqMan Universal PCR Master Mix, with AmpliTaq Gold and 0.625 *μ*l of the assay mix (forward and reverse specific primers and the specific probes), in concentrations optimised in preliminary reactions, in a total volume of 25 *μ*l. After thermal cycling, the instrument determined the allelic content of each sample in the plate by reading the generated fluorescence. A substantial increase in VIC fluorescence indicated homozygosity for wild-type allele, an increase in FAM fluorescence signalled homozygosity for mutant allele, whereas both fluorescence signals indicated heterozygosity.

### Calculations and statistics

Arithmetic means are given with s.e.m. Statistical significance of the difference between two groups was evaluated by Student's *t*-test. Differences were considered to be significant when *P*<0.05.

## Results

### BCRP expression in Caco-2 and WiDr HF- and LF-adapted cells

**Figure 1:**
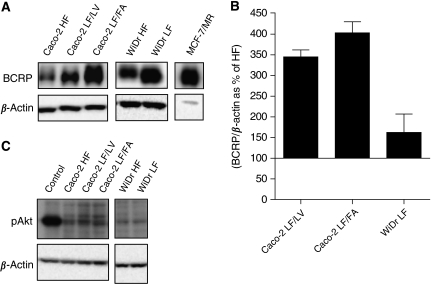
BCRP and pAkt expression in Caco-2 and WiDr HF- and LF-adapted cells and MCF-7/MR cells. (**A**) BCRP protein expression was determined by western blot analysis in Caco-2 and WiDr HF- and LF-adapted cells and MCF-7/MR cells. Per lane 15 *μ*g (Caco-2), 30 *μ*g (WiDr) or 1 *μ*g (MCF-7/MR) of protein was loaded. As a loading control, *β*-actin levels are indicated. (**B**) BCRP mRNA levels assessed by real-time LC-PCR were determined in Caco-2 and WiDr HF- and LF-adapted cells. Shown are the ratios between BCRP and the housekeeping gene, *β*-actin, presented as percent (%) of the HF cells. Bars represent the arithmetic means±s.e.m. of two independent experiments performed in duplicate. (**C**) pAkt protein expression was determined by western blot analysis in Caco-2 and WiDr HF- and LF-adapted cells. Per lane 30 *μ*g of protein was loaded. As a loading control, *β*-actin levels are indicated. Breast cancer resistance protein in Caco-2, data from Lemos *et al* (2008b).

We recently described the adaptation of Caco-2 cells, usually cultured in standard RPMI medium containing supraphysiological concentrations of folates (HF), to low-folate (LF) conditions (1 nM of LV or FA), resulting in the sublines Caco-2 LF/LV and Caco-2 LF/FA (Lemos *et al*, 2008b). Here we show that these LF-adapted cell lines had a 1.3–2.2-fold increase in the constitutive BCRP protein expression along with 3- to 4-fold increase in the mRNA levels (Figure 1A and B). Of note, despite the intracellular localisation of BCRP in Caco-2 cells, the LF-adapted cell lines displayed 4- to 6-fold resistance to a prototypical BCRP substrate mitoxantrone, suggesting that BCRP is functionally active in these cells (Lemos *et al*, 2008b). Another human colon cancer cell line adapted to LF conditions, WiDr LF, displayed only a moderate increase in the constitutive expression of BCRP protein and mRNA levels (1.6-fold) compared with their HF counterpart, as revealed by western blot and real-time LC-PCR (Figure 1A and B). Also in WiDr LF cells, BCRP has a predominant intracellular localisation; nonetheless, these cells were about twofold resistant to mitoxantrone compared with their HF counterpart, suggesting that also in these cells BCRP is functionally active (data not shown). In contrast with the colon cancer cell lines, MCF-7/MR breast cancer cells displayed overexpression of BCRP (Figure 1A), being predominantly located in the plasma membrane (Lemos *et al*, 2008b). It has been reported that the Akt phosphorylation status might affect the function and localisation of BCRP (Lemos *et al*, 2008a). Therefore, we investigated whether folate deprivation has an impact on Akt phosphorylation. For both WiDr and Caco-2, the levels of pAkt were similar in HF- and LF-adapted cells (Figure 1C), suggesting that cellular folate status has no impact in the phosphorylation status of Akt. Consistently Caco-2 and WiDr HF- and LF-adapted cells displayed a similar intracellular localisation of BCRP.

### Cellular growth inhibition with gefitinib and erlotinib in Caco-2, WiDr and MCF-7/MR cells

**Figure 2:**
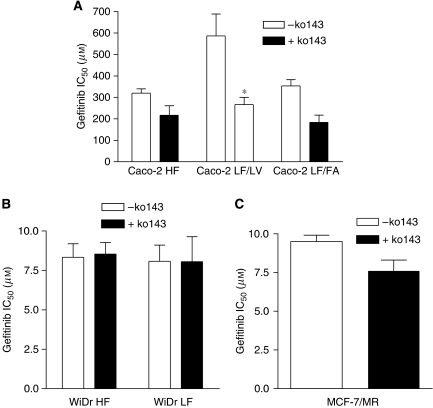
Cellular growth inhibition by gefitinib of Caco-2 and WiDr HF- and LF-adapted cells and MCF-7/MR cells. Growth inhibition by gefitinib was determined after 72 h of drug exposure in Caco-2 (**A**) and WiDr (**B**) HF- and LF-adapted cells and MCF-7/MR (**C**) cells. The BCRP-specific inhibitor Ko143 was added 15 min before the drug and it was present during the next 72 h at a concentration of 200 nM. Shown are the IC_50_ values, presented as arithmetic means±s.e.m., of at least three independent experiments. ^*^P<0.05 *versus* Caco-2 LF/LV–Ko143.

**Figure 3:**
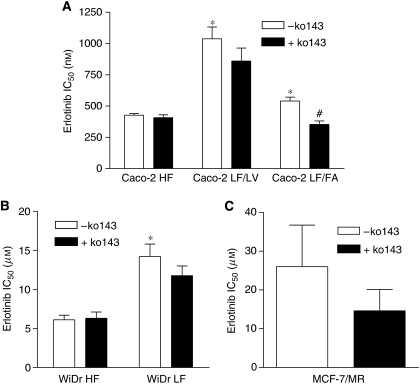
Cellular growth inhibition by erlotinib of Caco-2 and WiDr HF- and LF-adapted cells and MCF-7/MR cells. Growth inhibition by erlotinib was determined after 72 h of drug exposure in Caco-2 (**A**) and WiDr (**B**) HF- and LF-adapted cells and MCF-7/MR (**C**) cells. The BCRP-specific inhibitor Ko143 was added 15 min before the drug and was present during the next 72 h at a concentration of 200 nM. Shown are the IC_50_ values, presented as arithmetic means±s.e.m., of at least three independent experiments. ^*^P<0.05 *versus* HF cells. ^#^*P*<0.05 *versus* LF/FA cells–Ko143.

To investigate whether the different levels of BCRP expression in the Caco-2 and WiDr HF- and LF-adapted cell lines would have an impact in the anticancer efficacy of gefitinib and erlotinib, we performed growth inhibition studies in these cells as well as in the BCRP-overexpressing cell line MCF-7/MR. Caco-2 LF/LV cells showed 1.8-fold resistance to gefitinib and 2.4-fold resistance to erlotinib compared with their HF counterpart. Inhibition of BCRP with its specific blocker Ko143 (Allen *et al*, 2002) completely abrogated gefitinib resistance in Caco-2 LF/LV cells, but had almost no effect on erlotinib sensitivity (Figures 2A and 3A). In contrast, the Caco-2 LF/FA cell line displayed similar sensitivity to gefitinib and only a moderate level of resistance (1.3-fold) to erlotinib compared with the Caco-2 HF cells (Figures 2A and 3A). Interestingly, Ko143 rendered Caco-2 LF/FA cells 1.9- and 1.5-fold more sensitive to gefitinib and erlotinib, respectively (Figures 2A and 3A). No differences on gefitinib sensitivity were observed between the WiDr HF- and LF-adapted cell lines (Figure 2B). In contrast, WiDr LF cells were 2.3-fold resistant to erlotinib compared with their HF counterpart, a phenotype that was almost unaffected by BCRP inhibition with Ko143 (Figure 3B). MCF-7/MR cells were used in this study as control because they express high levels of BCRP and are highly resistant to BCRP substrates such as MR and methotrexate (Maliepaard *et al*, 2001; Ifergan *et al*, 2004). Breast cancer resistance protein was functionally active in these cells based on the observation that a BCRP-specific blocker, Ko143, could reverse MR sensitivity by 64-fold (data not shown). Nonetheless, BCRP inhibition by Ko143 induced only marginally decreases in the IC_50_ values for both gefitinib and erlotinib in these cells (Figures 2C and 3C).

### EGFR protein expression in Caco-2, WiDr and MCF-7/MR cells

**Figure 4:**
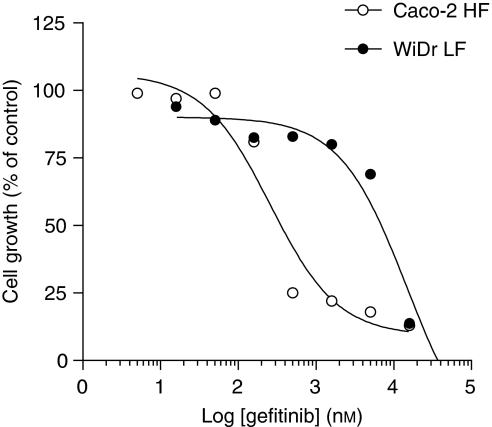
Epidermal growth factor receptor expression in Caco-2 and WiDr HF- and LF-adapted cells and MCF-7/MR cells. Epidermal growth factor receptor protein expression was determined by western blot analysis in Caco-2 and WiDr HF- and LF-adapted cells and MCF-7/MR cells. Per lane 40 *μ*g of protein was loaded. As a loading control *β*-actin levels are indicated.

Epidermal growth factor receptor protein expression is an important determinant of gefitinib and erlotinib sensitivity. Therefore, we investigated the expression levels of the receptor in all cell lines. Caco-2 cells, both HF and LF, displayed high levels of EGFR protein. In WiDr HF- and LF-adapted cells EGFR protein expression was markedly lower than in Caco-2 cells. Epidermal growth factor receptor was almost absent in MCF-7/MR cells (Figure 4).

### BCRP genotype in Caco-2, WiDr and MCF-7/MR cells

The BCRP C421A polymorphism was studied in Caco-2 and WiDr HF- and LF-adapted cells as well as in MCF-7/MR. All cell lines displayed the wild-type genotype (CC) (data not shown).

## Discussion

**Figure 5:**
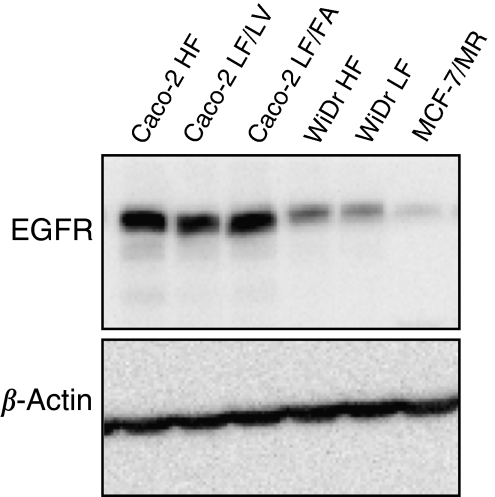
Representative dose/effect plot of cellular growth inhibition by gefitinib in Caco-2 and WiDr HF cells. The graph is representative of three independent experiments. s.d. <20%.

To study the impact of different levels of BCRP expression in the anticancer activity of two EGFR TKIs, gefitinib and erlotinib, we initially performed growth inhibition studies with these drugs in Caco-2 and WiDr HF- and LF-adapted cell lines as well as in MCF-7/MR cells, with MR-induced overexpression of BCRP (Maliepaard *et al*, 2001). Caco-2 LF/LV cells, but not Caco-2 LF/FA, showed 1.8-fold resistance to gefitinib compared with the HF cells. Blocking BCRP with its specific inhibitor Ko143 completely reverted the gefitinib resistance in Caco-2 LF/LV cells, suggesting that the resistance to gefitinib in these cells can be attributed to the increased expression of BCRP. Considering the higher BCRP expression in Caco-2 LF/FA over LF/LV cells, one would anticipate that these cells would also reveal resistance to gefitinib compared with the HF counterpart, but surprisingly this was not observed. This result might be explained by the limited range of drug concentrations (up to 1 *μ*M) within which BCRP is able to transport gefitinib (Elkind *et al*, 2005; Li *et al*, 2007). Owing to the higher expression of BCRP in Caco-2 LF/FA than in Caco-2 LF/LV cells, we would anticipate also a higher IC_50_ value for gefitinib, most likely above 1 *μ*M; however, at this concentration, gefitinib is no longer a substrate for BCRP, but rather an inhibitor (Li *et al*, 2007). Therefore, we hypothesise that gefitinib inhibits BCRP in Caco-2 LF/FA cells, thereby attenuating its expected levels of resistance in these cells. A similar complex interaction between BCRP and imatinib has been described by Nakanishi *et al* (2006) who showed that imatinib itself could attenuate its resistance by suppressing BCRP expression. Furthermore, Ko143 rendered Caco-2 LF/FA cells about twofold more sensitive to gefitinib, suggesting that BCRP does have a function in gefitinib sensitivity in these cells. In WiDr cells, no difference on gefitinib sensitivity was observed between the HF and LF cells, despite the higher expression of BCRP in the LF cells. Likewise, in MCF-7/MR cells, we did not observe major differences in gefitinib sensitivity when growth inhibition experiments were performed in the presence or absence of the BCRP inhibitor Ko143. Thus, although our results with Caco-2 cells strongly suggest that BCRP can actively extrude gefitinib and mediate resistance to this drug, the data obtained with WiDr and MCF-7/MR suggested that its function is highly variable. To further explore the mechanistic basis for this, we first investigated two parameters that could contribute to TKI resistance: (1) EGFR levels and (2) BCRP SNPs that may have a function in the observed TKI-resistant phenotypes. Interestingly, our present results were in line with observation by Yanase *et al* (2004) showing that when gefitinib-sensitive A431 lung cancer cells were transduced with BCRP (A431/BCRP) they became markedly resistant to gefitinib whereas BCRP transfection in gefitinib-insensitive leukemic K562 (K562/BCRP) and P388 (P388/BCRP) cells did not much further increase gefitinib resistance. This may be attributed to the fact that A431 cells express high EGFR levels to elicit high sensitivity, whereas K562 and P388 cells do not express EGFR thereby conferring inherent resistance to gefitinib. Consistent with this notion, we observed that Caco-2 cells displayed much higher expression levels of EGFR than WiDr and MCF-7/MR cells and thereby confer much greater sensitivity to gefitinib (Figure 5). Thus, conceivably, BCRP can be a major determinant in conferring gefitinib resistance in Caco-2 cells because they express EGFR and are more sensitive to the drug. Most likely, this is associated with the fact that gefitinib transport by BCRP is concentration dependent. It has been shown that low concentrations of gefitinib (<1 *μ*M) significantly activated BCRP-ATPase activity in isolated membranes of BCRP-expressing mammalian MCF-7/MX and A431 cells, whereas higher gefitinib concentrations (>1 *μ*M) had a markedly lower stimulatory effect (Elkind *et al*, 2005). Several SNPs in the *ABCG2* gene have been described that might have an important impact on BCRP protein expression and function (Yanase *et al*, 2006). Imai *et al* (2002) showed that the BCRP nonsynonymous SNP C421A was associated with markedly decreased levels of BCRP protein expression and also low levels of drug resistance. More recently, Cusatis *et al* (2006) reported that this polymorphism was associated with the occurrence of diarrhoea in patients receiving treatment with oral gefitinib. The authors suggested that the reduced protein levels and altered ATPase activity of the BCRP C421A variant might affect the oral absorption and/or elimination pathways of gefitinib thereby increasing the steady-state gefitinib plasma concentrations leading to the diarrhoea. In this context, we investigated the possible presence of this BCRP variant in the cell lines used in this study. However, all cells displayed the wild-type genotype (CC), suggesting that the discrepancy in our results could not be attributed to the C421A BCRP variant.

Caco-2 LF/LV cells were 2.4-fold resistant to erlotinib, compared with the HF cells. However, in this case, the BCRP-specific inhibitor Ko143 was not able to revert erlotinib resistance, suggesting that BCRP is not involved in erlotinib resistance in these cells. Consistently, Ko143 had no significant effect on erlotinib sensitivity in MCF-7/MR cells. In addition, WiDr LF cells were also 2.3-fold resistant to erlotinib, compared with the HF cells, and Ko143 had no effect on the resistant phenotype, corroborating the hypothesis that erlotinib resistance in LF-adapted cells is mediated by a mechanism independent of BCRP. Given the fact that erlotinib has been described as a BCRP substrate (Li *et al*, 2007), one could expect that this transporter would be implicated in erlotinib resistance. However, similarly to gefitinib, erlotinib is only transported by BCRP at low concentrations (⩽1 *μ*M), which can explain our observations in WiDr and MCF-7/MR cells, because these cells display IC_50_ values for this drug above 5 *μ*M. In Caco-2 cells, showing a considerably higher sensitivity to this drug, it is possible that the levels of BCRP overexpression in the LF-adapted cells do not reach a threshold that can confer erlotinib resistance. Of note, although Caco-2 LF/FA cells, which express the highest levels of BCRP among the Caco-2 cell lines, displayed only a marginal increase in erlotinib resistance compared with the HF counterpart, they became significantly more sensitive to the drug in the presence of Ko143. Therefore, we might envision a function for BCRP in erlotinib resistance, but its relative contribution is dependent on different factors determining intrinsic drug sensitivity of the cells and the levels of BCRP expression.

There are several potential mechanisms of resistance to EGFR inhibitors in tumour cells, including (1) target gene alterations and/or target protein loss; (2) loss/inactivation of downstream signalling molecules, activation of other tyrosine kinase receptor systems that are not EGFR related such as the insulin-like growth factor family of ligands and receptors; (3) activation of EGFR-independent tumour-induced angiogenesis, independent activation of intracellular signalling pathways that function downstream to the EGFR; and finally (4) molecular changes in cancer cells that affect EGFR inhibitor uptake (Bianco *et al*, 2005). In the present study, we investigated whether erlotinib resistance in the LF-adapted cells could be associated with a decrease or loss of EGFR protein. Western blot analysis revealed that in Caco-2 and WiDr cells EGFR was similarly expressed in the HF and LF cells, thus suggesting that erlotinib resistance is not mediated by a suppression or loss of EGFR expression.

It has been previously shown that folate supplementation/deprivation might have important therapeutic implications for several anticancer drugs (Hooijberg *et al*, 2006). In this study, we provide further proof for an impact of cellular folate status on drug efficacy, in particular for the EGFR TKIs gefitinib and erlotinib. Although the mechanism involved seems to differ for both drugs, our data clearly suggest that higher folate concentrations convey a beneficial impact in the activity of these TKIs. This is in agreement with a previous report from our laboratory, showing that folate supplementation triggered a reduced BCRP-mediated MR resistance in Caco-2 cells (Lemos *et al*, 2008b). Nonetheless, opposite effects have also been described (Ifergan *et al*, 2004), suggesting that the relationship between folates and drug resistance/efficacy, especially when associated with MDR-transporters, is complex and warrants further investigation.

In conclusion, our data suggest that BCRP is a major determinant of gefitinib resistance in Caco-2 cells with constitutively high EGFR expression. Conversely, in cells being intrinsically resistant to gefitinib due to low/absent expression of EGFR such as WiDR and MCF-7/MR cells, BCRP does not confer additional resistance. Rather than for gefitinib, our data suggested that BCRP is not a major determinant of erlotinib resistance in the cell models used in this study. Finally, we showed that modulation of extra/intracellular folate status might have an impact on gefitinib and erlotinib activity by mechanisms (in)dependent of BCRP functioning.
